# Poisoning by Colchicin

**Published:** 1890-11-01

**Authors:** 


### THE PRACTITIONER'S MIRROR AND RETROSPECT.

[*The Editor will be glad to receive offers of co-operation and contributions from members of the profession. There is no doubt that much valuable material full of interest and instruction is at present lost to science. This is largely so in the case of* (1) *the younger and less-known members of the hospital staff* (2) *those connected with cottage hospitals, and* (3) *the great body of medical practitioners not attached to any institution. The co-operation of all is cordially invited to enable the Editor to make* THE HOSPITAL *of the utmost possible use and value to the profession. Specialists willing to review books on their special subjects are invited to communicate this fact at once. All letters should be addressed to* THE EDITOR, THE LODGE, PORCHESTER SQUARE, LONDON, W.]

### MEDICINE.

### POISONING BY COLCHICIN.

Drs. P. Albertoni and O. Casali have described a case of poisoning by colchicin which must have been maliciously substituted for cotoin, 0·6 of which had been prescribed in divided doses three times a-day for obstinate chronic diarrhœa in a woman aged 43. Soon after she had taken the second dose, and about three hours after the first, she was seized with pains in the head, stomach, and abdomen, and general malaise, compelling her to take to her bed. When seen by a medical man four hours later she was suffering from severe abdominal pains, with violent vomiting and bloody stools and marked coldness of the extremities. She died with symptoms of gastro-enteritis 31 hours after taking the first dose. At the judicial autopsy there was found general hyperæmia of the intestinal mucous membrane and evidence of chronic catarrh, but no inflammation or ulceration of Peyer's patches. The heart was overlaid with fat, and the muscle fibres showed fatty degeneration and friability. The 22 remaining powders were amorphous, yellow, soluble in water, and with a bitter persistent taste, whereas cotoin is met with in the form of white crystals insoluble in water. The stock of the apothecary consisted of pure cotoin, but analysis showed the powders to be unmistakably colchicin, of which it happened that there was none in his shop. As colchicin is, under the regulations of the Italian Pharmacy Acts, not easily obtainable by the public, the conclusion was that the powders had been subsequently changed by some professional man with criminal intent.

C. Jacoby has been making some researches on colchicin, which has only recently been prepared in a crystallin form by Houdé and von Zeisse, in which the latter chemist states that it is combined with chloroform, the chloroform acting the part of a "water" of crystallization. The effect of this is to delay the manifestation of the symptoms, which, even after subcutaneous injection, do not appear for two or three hours. In experiments on cats and dogs these are nausea, vomiting, and diarrhœa. Next come changes in sensation and motility, indicating an ascending central motor paralysis. When the upper limbs have been involved in the paralysis, and the respiratory centre in the medulla is reached, death occurs from paralysis of respiration. the heart continuing to beat for twenty minutes after respiration has ceased. The *post-mortem* appearances are those of gastro-enteritis with ecchymoses and internal hæmorrhages.

The pure crystalline colchicin is almost inert on frogs, and even with mammals it appears that it must be converted into an oxidation product, which is the brownish amorphous body hitherto known in pharmacy, to which Von Zeisse would give the name of "oxydicolchicine." With frogs, 5 mgs. induce convulsions within half an-hour, and ultimately death from paralysis. Oxydicolchicine can be prepared from the crystalline colchicine by the action of strong acids, and the same oxidation is doubtless brought about in the bodies of warm-blooded animals, for the drugs act alike in equal doses, differing only in the time that elapses before the effects begin to be manifested.

